# Beyond the frontiers of neuronal types: fuzzy classification of interneurons

**DOI:** 10.1186/1471-2202-14-S1-P56

**Published:** 2013-07-08

**Authors:** Harold W Gutch, Demian Battaglia, Anastassios Karagiannis, Thierry Gallopin, Bruno Cauli

**Affiliations:** 1 Department of Nonlinear Dynamics, Max Planck Institute for Dynamics and Self-Organization (MPIDS), Göttingen, Germany; 2 Bernstein Center for Computational Neuroscience, Göttingen, Germany; 3 Department of Mathematics, Technische Universität München, Garching, Germany; 4 CNRS UMR 7102, Laboratoire de Neurobiologie des processus adaptatifs, Université Pierre et Marie Curie, Paris, France; 5 Neuroscience, Physiology and Pharmacology, Division of Biosciences, University College London, London, UK; 6 CNRS UMR 7637, Laboratoire de Neurobiologie et Diversité Cellulaire, ESPCI ParisTech, Paris, France

## 

Cortical neurons and, particularly, inhibitory interneurons display a large diversity of morphological, synaptic, electrophysiological, and molecular properties, as well as diverse embryonic origins. Various authors have proposed alternative classification schemes that rely on the concomitant observation of several multimodal features. However, a broad variability is generally observed even among cells that are grouped into a same class. Furthermore, the attribution of specific neurons to a single defined class is often difficult, because individual properties vary in a highly graded fashion, suggestive of continua of features between types. Going beyond the description of representative traits of distinct classes, we focus here on the analysis of atypical cells[1]. We introduce a novel paradigm for neuronal type classification, assuming explicitly the existence of a structured continuum of diversity. Our approach, grounded on the theory of fuzzy sets[2], identifies a small optimal number of model archetypes[3]. At the same time, it quantifies the degree of similarity between these archetypes and each considered neuron. This allows highlighting archetypal cells, which bear a clear similarity to a single model archetype, and edge cells, which manifest a convergence of traits from multiple archetypes.

A ready-to-use software package allowing classification of neuronal data with standard tools (MATLAB, Python, ...) via this fuzzy clustering approach without the need for a reimplementation of the algorithmic aspects is in preparation.
